# Carbon Capture and Utilization by mineralization of cement pastes derived from recycled concrete

**DOI:** 10.1038/s41598-020-62503-z

**Published:** 2020-03-27

**Authors:** Jan Skocek, Maciej Zajac, Mohsen Ben Haha

**Affiliations:** Global R&D, HeidelbergCement AG, Oberklamweg 2-4, 69181 Leimen, Germany

**Keywords:** Civil engineering, Structural materials

## Abstract

Reduction of CO_2_ emissions associated with cement production is challenging in view of the increasing cement demand and the fact that major part of the emissions originates from the main raw material used - limestone - which can be only to extremely low amount substituted. A Carbon Capture and Utilization (CCU) approach based on mineralization of fines derived from concrete appears to be a viable alternative to reduce these emissions. The CO_2_ sequestration and the reactivity of the obtained carbonated recycled fines is experimentally demonstrated for lab as well as industrial materials for different mineralization conditions. It is shown that all CO_2_ originally released by limestone calcination during clinker production can be sequestered by the full carbonation of the fines within a short time. Upon full carbonation, gels with pozzolanic properties form in the fines irrespective of the conditions tested. The carbonated fines have specific CO_2_ savings more than 30% higher than the simple clinker replacement by limestone.

## Introduction

Global urbanization and economic development increase demand for new buildings and infrastructure and hence for concrete. Already now, concrete is the second most used substance by mass after water. Even though concrete has low specific CO_2_ emissions below 150 kg_CO2,eq_/ton concrete^1^, its abundance makes it responsible for 5–8% of man-made CO_2_ emissions^1–4^. Most of the concrete’s emissions result from production of the main cement component, the clinker. Hence, focus of researchers as well as of cement producers has been on lowering the clinker content in cements and on improving the efficiency of clinker production. As summarized in^1^, both these approaches have already reached their potentials in mature economics. Almost all suitable Supplementary Cementitious Materials (SCMs) available are already used in cement and concrete production. For the clinker production, further energy efficiency improvements would have only a minor effect as the energy consumption approaches the lowest theoretical value and is often driven by additional objectives such as alternative fuels usage rate.

Nevertheless, the cement industry needs to drastically reduce its emissions and at the same time satisfy the increasing cement demand of the global economy^5^. At the level of concrete structures and concrete applications, several approaches can bring considerable CO_2_ savings. These include re-usable structures with extended service life or concretes with reduced cement contents^3,6^. At the level of cement production, extended use of calcined clays and limestone as SCM are the only approaches applicable at large enough scale. With the ongoing departure from coal-fired power plants and decreasing demand for pig iron in mature markets, the availability of blast furnace slags and fly ashes is expected to decrease. Calcined clays and limestone have the potential to replace these SCMs in the current market as well as provide sufficient volumes of SCMs in growing markets. However, clinker replacement by limestone or calcined clay is limited by the performance and compositional constrains. Limestone alone can be used at replacement levels up to ~20% without negatively affecting cement performance (Palm *et al*., 2016^7^ and references therein). Combination of calcined clay with limestone can replace up to 50% of clinker in a cement^3^. However, early strengths are impaired limiting applicability of such cements to specific applications such as precast concrete and a high quality clay is required for appreciable strength development enabling the clinker replacement of 50%^8^. The use of limestone and calcined clays will not dramatically reduce the average clinker factor of the future cements. Hence, the overall CO_2_ saving potential will be limited.

At the level of clinker, no better alternative to Portland clinker satisfying both the technical requirements as well as the market volumes is globally available^1^. Since the major part of the CO_2_ emissions associated with Portland clinker production comes from limestone calcination and since there are no other suitable calcium sources than calcium carbonates at large scale, Carbon Capture and Storage (CCS) of the CO_2_ released during the clinker production is the only suitable approach for a substantial CO_2_ emissions reduction^6^.

According to IPCC^9^, the CCS is defined as capture of CO_2_ from point sources combined with its geological storage. In some sectors, CCS is cost-effective in reducing emissions, but there are significant gaps: high capital costs, uncertain potential storage capacity, uncertain long-term impacts and stability of the storage sites, increasing public resistance to CCS, energy costs and the related indirect CO_2_ emissions. A potentially suitable alternative to the geological storage is the mineral carbonation^10,11^, also called mineralization, i.e. the concept of storing CO_2_ in the form of calcium and magnesium carbonates^12^. Calcium and magnesium carbonates are poorly soluble in water^13^ and are environmentally friendly minerals that could provide a permanent CO_2_ storage solution. In the mineral carbonation, the CO_2_ (captured) reacts with calcium or magnesium originating from rocks rich in alkaline earth silicates such as e.g. olivine (MgSiO_4_) and wollastonite (CaSiO_3_)^14–16^. On geological time scales, these silicate rocks react with CO_2_ during the natural weathering to form the corresponding carbonates and SiO_2_^17^. As a concept for CCS, the reaction needs to be accelerated which requires mechanical, thermal and/or chemical treatment and high pressure for the reaction. These steps are the major challenge for the large-scale industrial deployment of the CO_2_ mineralization^18^. Another challenge related to the mineralization based on silicate rocks are their large volumes required, the associated mining activities and transport of the rocks and/or CO_2_ to the carbonation plant. Depending on the materials used, it is necessary to have between 1.6 to 3.7 t of the carbonatable materials to fix 1 t of CO_2_. For each ton of clinker produced, approximately 1.6 t of limestone is quarried and 0.865 t of CO_2_ emitted (“The Cement Sustainability Initiative,” 2017). Applying the silicate rocks mineralization to capture all the CO_2_ emitted would increase the mining activities associated with the clinker production 2–3 times and so would the environmental impacts. After the CO_2_ fixation, the products need to be used or disposed. Due to the large volumes produced, there is no market large enough for products of the mineralization, which is a mixture of the carbonates and SiO_2_. Separating these two products and using them in value-added applications can improve the economics of a given single installation, but cannot substantially contribute to the overall reduction of CO_2_ emissions because of the small market size for these value-added applications.

In this paper, an alternative approach of the (CCU) based on mineralization of fines derived from concrete recycling and their utilization in cement production is proposed. Its key assumptions are experimentally verified and the overall impacts discussed.

## Materials and methods

The industrial recycled concrete fines contain several constituents^19^. These include hydrated cement paste, un-hydrated components of the original cement and fines originating from the sand and coarse aggregates. As even a single demolished structure contains several concrete types and a recycling facility treats concretes from several demolition sites, the composition of industrial fines is complex and highly variable. Additionally, the composition of the original concretes is not known. To overcome these difficulties, synthetic hydrated cement pastes were prepared. This enabled control over the composition of the cement pastes as well as quantitative analysis of the results obtained.

Portland cement CEM I 42.5 R according to EN 197-1 containing Portland cement clinker, calcium sulfate and a small quantity of limestone was hydrated for 3 months at 40 °C and w/b = 0.4. The elevated curing temperature was used to accelerate the hydration process in order to simulate the old cement paste from demolished concrete. The hydration degree of the cement clinker was close to 90% after the curing. One notes that hydration at 40 °C does not significantly modify the cement paste but considerably accelerates the hydration when comparing to 20 °C^20,21^. After the hydration, the samples were ground to two different fractions: to pass the 90 µm sieve and crushed below 2 mm, respectively. Both these fractions were then carbonated. Batch carbonation experiments were performed in an autoclave (Berghof BR4000). The starting CO_2_ pressure ranged from 1 to 8 bars and the tested temperature ranged between 20 and 200 °C. For these experiments, the autoclave was loaded with the cement paste sample stored above distilled water and preloaded with pure CO_2_ up to the starting pressure. Furthermore, the autoclave was heated to the targeted temperature and hold at for 1 hour. Afterwards, the autoclave was let to cool down (without active cooling) to room temperature. The experiments were designed that each of them took about 15 hours independently on the temperature studied. After the experiment, the carbonation content was evaluated by comparing the CO_2_ bound in carbonates retrieved from thermal gravimetry with the Steinour’s formula^22^ for the theoretical CO_2_ uptake. Thermogravimetric analyses were done using NETZSCH STA F449 on 30 ± 2 mg of a ground material in open vessels in N_2_ atmosphere at a heating rate of 20 °C/min up to 1050 °C.

**Figure 1:**
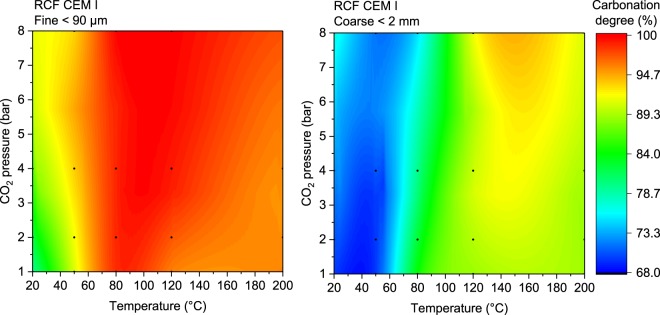
Map of carbonation degree of the mimic recycled concrete fines (RCF) based on CEM I depending on the initial CO_2_ pressure and carbonation temperature. The points indicate the measurements points. The map was prepared by interpolation with the ORIGIN software.

Results presented in Fig. 1 revealed that temperature as low as 20 °C is enough to carbonate cement pastes to a high extent when fine materials is used. At 80 °C, close to full carbonation of the samples was achieved for the finer materials. The pastes carbonated at 80 °C and starting pressure of 8 bars were chosen for the further study. The carbonated samples were assessed by a multi-technique approach comprising thermodynamic modelling as well as traditional experiments such as thermogravimetric analyses, FTIR, X-ray diffraction and scanning electron microscopy coupled with EDX mapping.

Thermodynamic modelling was carried out using the Gibbs free energy minimization program GEMS v3.3^23,24^. The thermodynamic data for aqueous species, solids and gas phases include the PSI-GEMS thermodynamic database^25,26^ and cement specific data base^27^ including some zeolites^28,29^. To simulate the effect of the carbonation of the cement pastes, for the thermodynamic calculation, the cement pastes were titrated with the pure CO_2_. The calculations enable predicting the progressive modification of the phase assemblage during the carbonation experiment as well as the effect of the temperature. The calculations were performed at temperature of 20 °C and 80 °C and at pressure equal to 8 bar. The calculations at the two temperatures are needed since in the experimental setup, the carbonation starts at 20 °C and continues at progressively higher temperature up to 80 °C. For the calculations, it was assumed that the hydration degree of the cement clinker does not change.

The quantitative phase composition of the clinkers and cement pastes was examined using the XRD analysis coupled with the Rietveld refinement method. The XRD patterns were obtained at room temperature (24 ± 2 °C) using a Bruker D-8 Advance in a θ-θ configuration with CuKα radiation (λ = 1.54059 Å) and equipped with the LYNXEYE (1-d) detector. The generator settings were 40 kV and 40 mA. The measurement range was 5 ° to 70 °2θ with a step-size of approximately 0.02 °2θ. Rietveld analysis was carried out on *in-situ* samples (not stopped pastes) by using the external standard method, with a slice of quartzite rock as secondary standard calibrated against NIST corundum. Additionally, the hydration-stopped samples were blended with an internal standard (10 wt.-% zincite). For these samples, the Rietveld calculations were carried out giving similar results as the external standard method. Consequently, these results are not shown here. For the hydration stopping, the solvent exchange method using isopropanol and diethyl ether^30,31^, was used.

TGA was used to quantify the bound water (BW) and portlandite (CH) contents of the stopped samples. The sample weight at 850 °C was used to normalize the results to the initial binder mass.

The FTIR-ATR data were obtained sing the device Perkin Elmer Spectrum 100. The frequency range was 500–4000 cm^−1^.

For SEM investigations, unground cement paste samples were carbonated in form of 3 mm thick discs. The disc was then stored 24 h in isopropanol, dried and stored in a desiccator with silica gel beads and under vacuum for another 4 days to evacuate the isopropanol. All samples for SEM investigations were impregnated under vacuum with a spectrally transparent epoxy resin (EpoTek® 301), gradually polished down to ¼ μm with a diamond spray and petrol as a lubricant and coated with a thin conductive layer of carbon (10 nm). The scanning electron microscope used for this study was Zeiss EVO LS10 equipped with Quantax400 Detector from Bruker.

Finally, to assess the pozzolanic reactivity, blends of the carbonated pastes with portlandite were prepared and investigated. This allowed information about the kinetics of the pozzolanic reaction and its products to be gained and hence the performance of the material in cements to be assessed^32^ [p. 3]^33^. The blend contained 60 wt.-% of the carbonated paste, 39 wt.-% of portlandite and 1 wt.-% of NaOH. It was mixed with water at water to solids ratio of 0.6 and stored sealed at room temperature (20 ± 0.5 °C). The reactivity was assessed using the same techniques as applied for the carbonated paste characterization using hydration-stopped and ground samples.

## Results and interpretation

### Carbonation of cement paste

**Figure 2:**
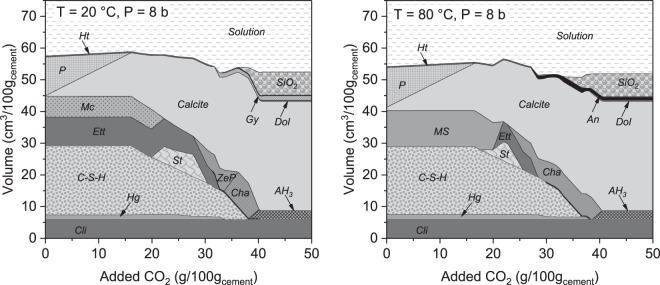
Modeling changes of the phase assemblage during the carbonation process at 20 °C (left) and 80 °C (right) and pressure of 8 bars. Cli – unreacted cement Portland clinker incl. limestone, Hg – siliceous hydrogarnets, C-S-H – C-S-H phase, Ett – ettringite, Mc – monocarbonate, MS – monosulphate, P – portlandite, St – strätlingite, Gy – Gypsum, An – anhydrite, Dol – dolomite, AH_3_ – alumina hydroxide, SiO_2_ – amorphous silica oxide, ZeP – Zeolite P(Ca) (CaAl_2_Si_2_O_8_·4.5H_2_O), Cha – Chabazite (CaAl_2_Si_4_O_8_·6H_2_O).

Thermodynamic modeling (GEMS software) was carried on to investigate the changes of the phase assemblage during the cement paste carbonation. The modelling results are presented in Fig. 2. The hydrated non-carbonated cement paste contains the expected^34–36^, hydrates: C-S-H phase, portlandite, ettringite, monocarbonate (note that the starting cement contained limestone) and small quantities of hydro garnets and hydrotalcite. Increasing the temperature to 80 °C has an important impact on the phase assemblage. At the temperatures higher than about 50 °C, the monocarbonate is no more a stable phase^20,37^. This results in ettringite decomposition and formation of monosulphate. Independently of the temperature, the progressive carbonation has a similar effect on the phase assemblage: firstly, portlandite is carbonated, then AFm phases followed by ettringite and finally the C-S-H phase. Products of the carbonation are initially calcite and temporary hydrates like strätlingite and zeolites. The main final products are calcite, silica, aluminium hydroxide and calcium sulfate, in agreement with the literature data^29^. The complete carbonation of the samples is achieved when about 40 g CO_2_/100 g_starting cement_ is added.

**Figure 3:**
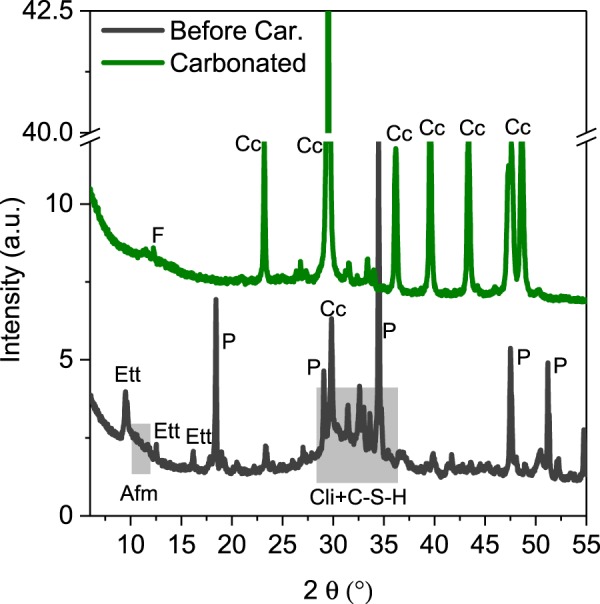
XRD data of the cement pastes before and after carbonation. Main effects of ettringite (Ett) AFm phases (AFm), portlandite (P), calcite (Cc), clinker (Cli), C_4_AF (F) and C-S-H (C-S-H) phases are highlighted.

**Figure 4:**
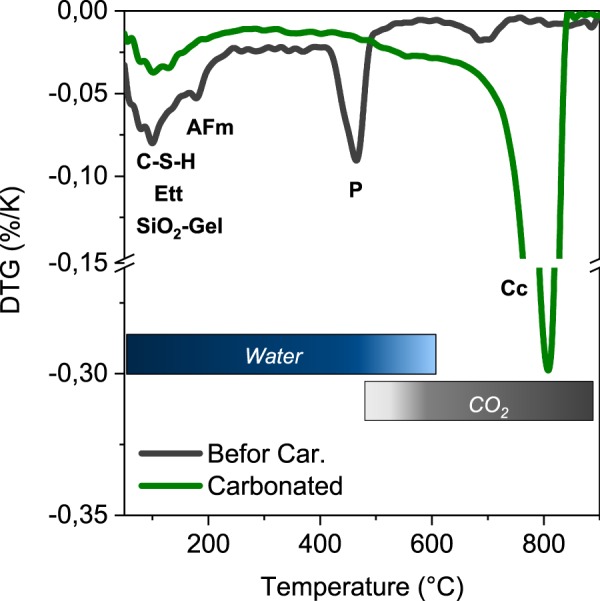
DTG data of the cement pastes before and after carbonation. Main effects of ettringite (Ett) AFm phases (AFm), portlandite (P), calcite (Cc),C-S-H phase (C-S-H) and SiO_2_ gel are highlighted.

The results of the thermodynamic modelling were compared to the experimental results. Figures 3 and 4 depict the XRD and TGA data of the hydrated paste before and after the carbonation. In agreement with the thermodynamic modeling, the main hydrates are ettringite, portlandite, AFm and the C-S-H phases. A part of the unreacted cement clinker is also visible. After the carbonation, the main crystalline phase is calcite. Rietveld calculations indicate that also C_3_S and C_2_S have completely reacted during the carbonation, while the C_4_AF phase did not show any additional reaction. Besides calcite, small quantities of vaterite and aragonite are present. The other carbonation products cannot be characterized based on these data, apart from the fact that they are XRD-amorphous. However, the broad effect between 50 and 300 °C indicates presence of hydrated gels. The content of calcium (magnesium) carbonates according to TG after the carbonation is about 95 g/100 g_starting cement_ corresponding to about 42 g of CO_2_ bound. This indicates close to complete carbonation of the paste. The bound water content calculated as the mass loss between 50 and 350 °C corresponds to 8 g/100 g_starting cement._

**Figure 5:**
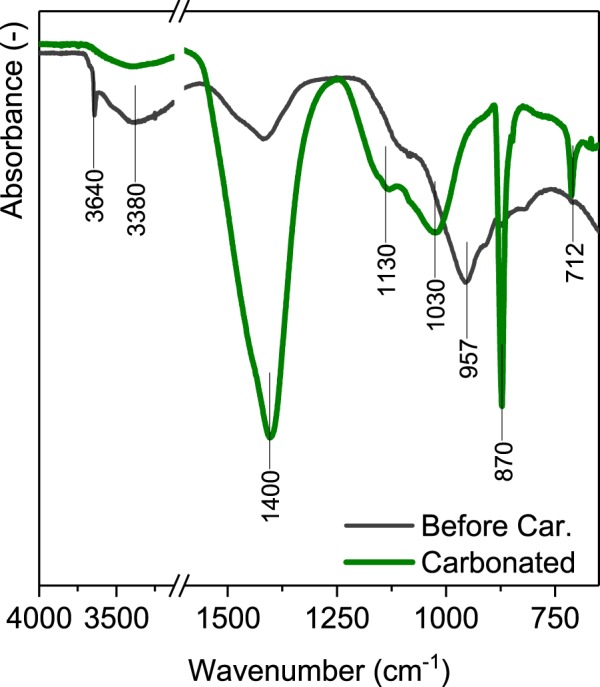
FTIR spectra of the hydrated cement pastes before the carbonation and after the carbonation.

The FTIR technique was used to investigate the structure of the amorphous gels formed during the carbonation process.Results are presented in Fig. 5. The FTIR spectra of the hydrated cement paste are characterized by a large signal between 800 and 1200 cm^−1^ with the peak at 957 cm^−1^ which may be associated with the asymmetric stretching vibration ν_3_ of Si-O bonds^38,39^. Similar data were obtained for hydrated C_3_S pastes^40,41^, where the peak between 967 and 954 cm^−1^ was associated with the asymmetric stretching vibration of Si-O bonds in the C-S-H gel Q^2^ units. For the carbonated sample, this effect significantly decreases and the peak shifts to 1030 cm^−1^. This is associated with the carbonation-induced decalcification of the C-S-H phase, since it is reported that the ν_3_ band shifts to the higher wave number with the increasing silica polymerization^38^. The same results were observed for the hydrated and then fully carbonated C_3_S pastes, where the main carbonation products was hydrated silica^41^. Consequently, the FTIR data confirm the presence of amorphous silica gel in the sample investigated after the carbonation.

The FTIR spectrum has a wide band at around 3380 cm^−1^ generated by O-H bonds stretching from water in hydrates and from water absorbed on their surfaces. It is noticeable that content of water in the carbonated sample is significantly lower, in agreement with the TG data (Fig. 3). The clearly visible effect at 3640 cm^−1^ is associated with OH groups in portlandite^39^.

Finally, the FTIR spectra confirms that the calcite is the main polymorph of the calcium carbonate present in the carbonated system with the effects at 712 cm^−1^, 870 cm^−1^ and 1400 cm^−1^ ^39^.

The identification of alumina containing phases is challenging since the vibration bands of Al-O bonds overlap with the Si-O signals. Because the Si content is higher than the Al content in the cement investigated, the bands in the range 800–1200 cm^−1^ are dominated by the silicates. Additionally, according to the XRD results, the alumina bearing phases are as well amorphous, which results in a broadening of the effects. However, it is noticeable that the alumina hydroxide, predicted to be main alumina bearing phase in the carbonated sample, has the main absorption bands in the region of 1030 cm^−1^ ^39^. The peak at 1130 cm^−1^ can be associated with the asymmetric stretch vibration modes of SO_4_ tetrahedra^39^.

**Figure 6:**
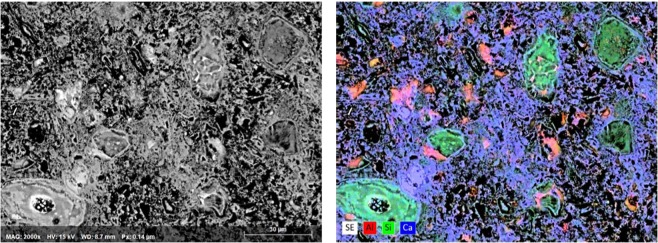
SEM-BSE micrograph (left) and EDS map (right) of the carbonated cement paste.

The backscattered electron image from the carbonated paste and the elemental map of the main elements are shown in Fig. 6. The black areas in both images correspond to porosity. The dark grey areas rich in silicon are associated with the decalcified C-S-H phase, which according to the FTIR study and thermodynamic modelling is a silica gel. Additionally, some dark areas are rich in alumina. These areas mostly correspond to space initially occupied by cement grains. The brighter gray regions comprise calcite intermixed with other carbonation products. The morphology of the bright grey areas indicates that the calcite precipitates only in the regions occupied by the outer product, i.e. the space initially occupied by the mixing water^42^. The brightest regions that are in general rich in Al are unreacted C_4_AF grains. Note that the data for Fe were omitted for better readability. Generally, Fe remained in the original clinker grains regains also rich in Al.

### Pozzolanic reactivity and properties of the re-carbonated paste

Characterizations of the carbonated samples revealed that the main components are calcium carbonate and amorphous silicate and alumina gels. Pozzolanic reactivity of these gels would make the carbonated samples suitable for clinker replacement as supplementary cementitious materials during the cement production^43^.

**Figure 7:**
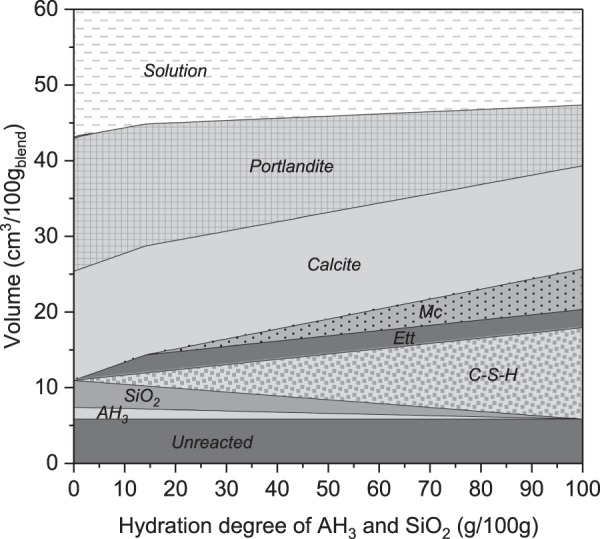
Thermodynamic modelling of the hydration of the carbonated paste in the presence of portlandite.

Thermodynamic modelling was applied to predict the evolution of the phase assemblage during the reaction of the carbonated cement paste with portlandite. The same model as above was used. However, instead of adding CO_2_, the hydration degree of alumina hydroxide and silica gels were gradually increased. The reaction of SCMs, calcium sulfate and calcite was not restricted, i.e. calcite and gypsum could freely dissolve or precipitate, depending on the system thermodynamic state. As a staring material, the fully carbonated cement paste from Fig. 2 was taken. However, the magnesium and iron bearing phases were neglected. Results of the modeling are shown in Fig. 7. The modelling indicates that during the reaction, three main phases are formed: the C-S-H phase, ettringite and, once the calcium sulfate gets depleted, monocarbonate. The model indicates that the carbonated paste is fully reacted when about half of the portlandite added is consumed.

**Figure 8:**
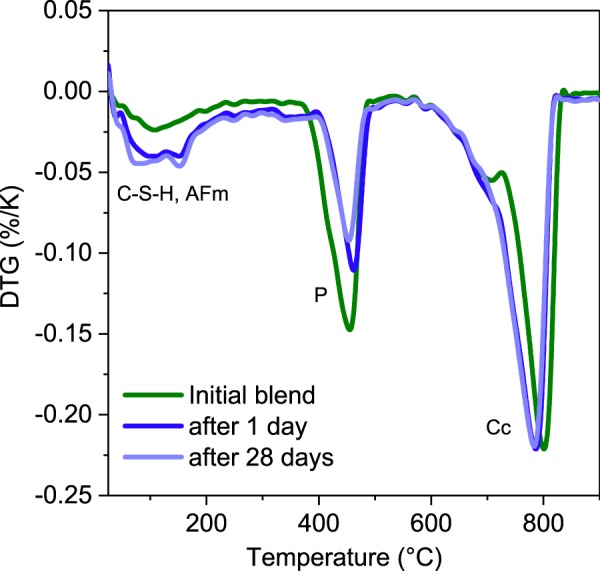
Results of the thermogravimetric investigations. The main effect of C-S-H (C-S-H) phase, AFm (AFm phases, mainly hemicarbonate and monocarbonate), P (portlandite) and Cc (calcite) are marked.

**Figure 9:**
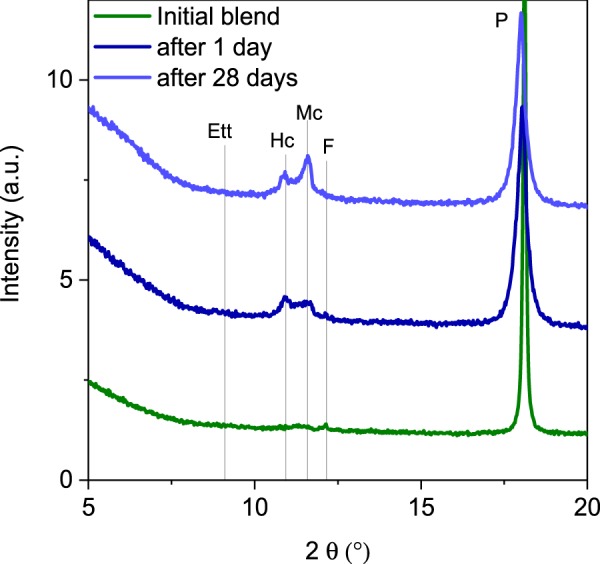
XRD results in the range 5 to 20 ° 2θ where the main AFm hydrates (Hc hemicarbonate and Mc monocarbonate) are visible as well as portlandite (P). Reflexes of ettringite are not present.

The DTG data obtained after 1 and 28 days of mix hydration are compared to the initial blend before mixing with water in Fig. 8. The DTG data show portlandite consumption accompanied by formation of C-S-H and AFm phases. The calcite content does not change significantly as also predicted by the thermodynamic modeling. The shape of effects in the range of 50–150 °C on the DTG curves indicates that ettringite is not present or its content is low^33,44^. This is confirmed by the XRD data shown in Fig. 9 where the presence of the AFm phases like hemi- and mono-carbonate is shown. However, reflexes associated with ettringite are not visible. These data may indicate that the calcium sulfate present in the carbonated cement paste is not reactive. Alternatively, the formation of an amorphous ettringite with untypical TG pattern cannot be excluded as well as absorption of sulfates on the gels with high specific surface areas. Nevertheless, the origin of this phenomenon is currently not explained and is under investigation.

**Figure 10:**
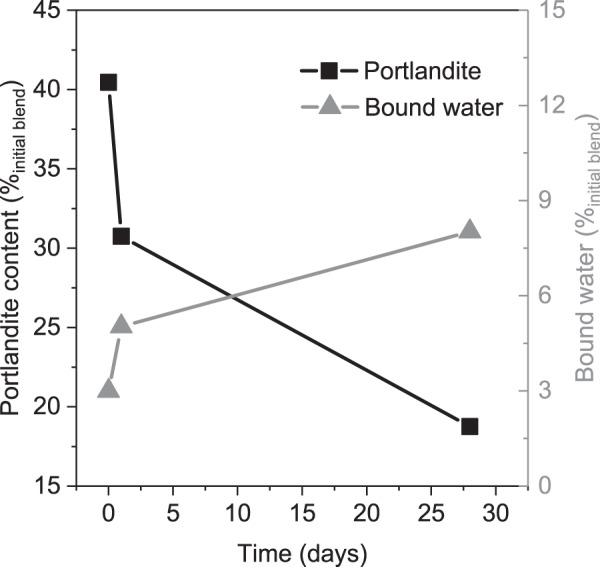
Evolution of the bound water, and portlandite contents over the hydration time.

The bound water evolution shown in Fig. 10 indicates a progressive reaction. Note that the bound water content was measured between 50 and 350 °C, i.e. it does not include the water bound in portlandite^32^ [p. 3]. The bound water content is significantly lower when compared to a hydrating cement that is in the range of 20 g/100 g_dry cement_, thought, it is well in the range for similar pozzolanic systems^33,44,45^. The portlandite content is decreasing in parallel to the increasing bound water content. It is noticeable that about a third or half of portlandite is consumed after 1 and 28 days of hydration, respectively. When comparing to the thermodynamic modelling results, the portlandite content at 28 days indicates a complete reaction of alumina and silica gels. Already at one day, the reaction degree of 60~70% is achieved. This in turn demonstrates that the reaction of the investigated material is rapid when compared to typical SCMs like slag and fly ash^33,44,45^ and similar to the hydration kinetics of metakaolin^46–48^.

### Re-carbonated paste as cement constituent

**Figure 11:**
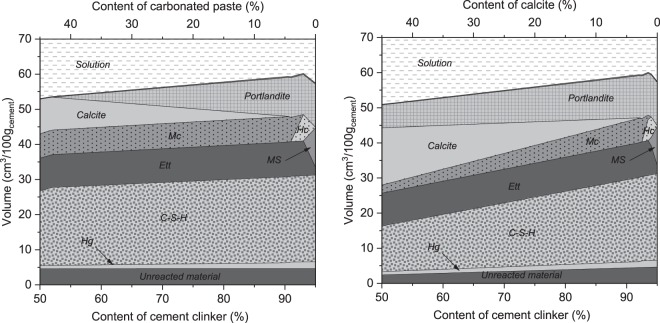
Thermodynamic modelling of the hydration of the carbonated paste in cement (left) compared to calcite addition (right).

The impact of the carbonated paste on the hydrates assemblage in a composite cement comprising cement clinker, the carbonated paste and 5% of anhydrate was predicted by the thermodynamic model as well. The same clinker composition and degree of hydration as used in the initial paste in Fig. 2 were assumed. Complete hydration of alumina and silica from the carbonated paste was assumed, in agreement with the experimental data at 28 days. Iron, sulfate and magnesium bearing phases in the carbonated paste were assumed to be inert since their reactivity could not be deduced from the experiments performed. The results are compared to the calcite addition in Fig. 11. Both hydrates assemblages are qualitatively comparable down to ~55% clinker content and show the aforementioned effect of carbonates on stabilization of ettringite demonstrated by the maximum of the solid volume at around 2.5% replacement^34,49^. However, there are significant quantitative differences. The pozzolanic reaction of the carbonated paste results in portlandite consumption and increased formation of the C-S-H phase and of monocarbonate. The carbonated paste-containing cement is expected to have higher strength due to the higher C-S-H content than its limestone equivalent^47,50^. At clinker contents below ~55%, no more portlandite is available for the pozzolanic reaction and the C-S-H content decreases comparably to the limestone addition with further clinker replacement.

According to the TG results discussed before, the carbonated paste bound 42 g of CO_2_ per 100 g of the initial cement, which corresponds to 28 g of CO_2_/100 g of the carbonated paste. Replacing clinker with average CO_2_ emissions of 86.5 g of CO_2_ per 100 g of clinker^51^, the overall CO_2_ saving sums up to 114.5 g of CO_2_ saved per 100 g of clinker replaced, disregarding any indirect emissions. Such saving is more than 30% higher than the limestone replacement while the performance of the cement with carbonated paste is expected to be better.

## General discussions

Concrete structures are demolished at the end of their service life. In the past, the concrete rubble was dumped. However, significant effort has been dedicated to further reuse the demolished concrete. Today, reuse of demolished concrete has become a common practice. The recent development of the concrete recycling technology allowed the shift from a simple size-reduction needed for the reuse in e.g. road construction towards a real concrete recycling, i.e. towards using the recycled materials again in concrete production. Multiple concrete recycling technologies capable of separating coarse aggregates are applied nowadays^52,53^. Nevertheless, the potential of the finer fraction left is not yet fully utilized. This fraction, called recycled concrete fines, contains the hardened cement paste together with fines from sand and aggregates.

The hardened cement paste comprises cement hydrates rich in calcium that are able to react with CO_2_ present in the atmosphere and permanently bind CO_2_ into calcite, the main component of limestone originally used for cement production. Theoretically, all the CO_2_ released from limestone during calcination (material CO_2_) can be bound back by the hardened cement paste. In reality, less than half of the material CO_2_ is bound during the structures lifetime and subsequent demolition since the carbonation rate is low^54,55^. Hence, concrete after its service life represents a globally significant potential CO_2_ sink, which is not utilized.

Applying carbonation to concrete production and recycling is not new. Recent studies focused on two main domains of concrete carbonation. The first one is the treatment of the recycled concrete aggregates with CO_2_ in order to improve their properties enhancing their recycling rate in concrete^56–58^. The second one concerns the CO_2_ treatment of cement-based materials in order to accelerate the evolution of the performance e.g.^59–63^ including the binders based on special, low hydraulically active clinkers^41,64,65^. Lately, some authors suggested that the recycled concrete fines can be valorized by carbonation for the use in the concrete production^66,67^. Studies on the carbonation of the hydrated cement pastes revealed that carbonation is occurring at mild conditions, i.e. temperatures in the range of 20–90 °C and pressures in the range of 1–10 bars. At this temperature and pressure, it is possible to carbonate between 10 and 90% of the carbonatable calcium as has been shown in this study. Generally, the higher the pressure and temperature, the higher carbonation extent and kinetics^10,58,67–69^. In contrary, high pressures of 150 bars or higher and reaction temperatures around 150 °C and higher^11^ are needed to achieve a comparable carbonation degree in natural magnesium and calcium silicate rocks.

**Figure 12:**
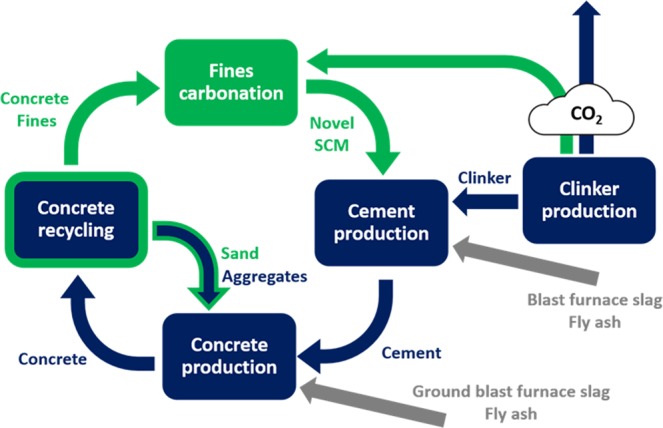
Scheme of the production of the cement characterized by substantially lower CO_2_ emissions. The green color highlights improvements of the process compared to the current situation shown in blue. The gray color highlights the traditional supplementary cementitious materials input with uncertain future availability.

The proposed approach in this study combined concrete recycling with the CCU by using fines derived from concrete recycling and valorizing them by carbonation. The process is schematically shown in Fig. 12 and has several advantages.

The advanced recycling technology enables an appropriate separation of the sand and paste. This facilitates re-use of the sand fraction for the concrete production and the clean paste can be efficiently used for the carbonation. This helps preserving natural resources. Assuming that most of demolition sites are close to newly built structures, the need of sand and gravel transportation is also limited. The recycled concrete fines carbonate readily, which enables a direct usage of exhaust gases from the cement plant. Hence, the process of fines carbonation can be used as CO_2_ capture and mineralization in a single step. Furthermore, the carbonated cement paste may be used as a SCM provided it is completely carbonated. This in turn further reduces the use of natural resources and enables further reduction of the clinker content in a cement and hence further reduction of the CO_2_ emissions.

However, achieving the target and closing the CO_2_ loop has two crucial prerequisites. The hydrated cement paste needs to be properly separated from coarse aggregates and sand during concrete recycling, going beyond possibilities of commonly available technology. Appropriate technologies still need to be developed and implemented at relevant scale. These activities are not discussed within this contribution. The second prerequisite is that the hydrated cement paste needs to bind all the material CO_2_ released during production of the cement it originally contained and the carbonated paste need to be a reactive material suitable for cement production. In this paper, the proof of concept for the second prerequisite was presented. Hydrated pastes are fully carbonated in a technologically feasible time and its properties including the pozzolanic reactivity characterized. Further details are provided in a series of our recent publications^70^.

## Conclusions

In this paper, a new approach to CCU based on mineralization of fines derived from concrete recycling and their utilization in cement production was proposed and its key assumptions experimentally verified and discussed. It could be demonstrated that:

- The hydrated paste can be re-carbonated within a short period and under conditions that are significantly milder compared to those used for carbonation of silicate rocks.
- The carbonated paste comprises mainly calcite and alumina and silica gels.
- The alumina and silica gels in the carbonated paste are highly reactive, achieving complete reaction within 28 days of hydration, which makes them suitable materials for clinker replacement in composite cements.
- The carbonated paste is a suitable supplementary cementitious material with CO_2_ saving potential up to 114.5 g of CO_2_ per 100 g of the carbonated paste, which is more than 30% higher than the savings achieved by limestone replacement.
- Up to 30–40% of clinker can be replaced by the carbonated paste in cement.

The principal feasibility of the approach could be demonstrated. Still, there are several open points requiring further research such as destiny of sulfate in the carbonated paste and reactivity of iron and magnesium bearing phases. Further detailed investigations focused on carbonation conditions and kinetics as well as on impact of the carbonated paste addition on cement and concrete properties will be reported.

As a CCU approach, the mineralization of fines derived from concrete recycling and their utilization in cement has several advantages compared to the CCS with geological storage as well as compared to the storage by mineralization of silicate rocks:

- The approach can be realized within the construction sector since the carbonatable materials come from demolished concrete and the carbonated paste comprise a part of cement used in new concrete.
- The approach contributes to the circular economy and preserves natural resources as all materials derived from demolished concrete are recycled back to concrete.
- The conditions for fines carbonation are mild compared to those needed for silicate rocks and are close to the conditions in the stack of a cement plant.
- The logistics involved in transporting the concrete fines to the cement plant is offset by the saved transport of sand and aggregates assuming that the demolition site is closer to the new construction site than a quarry, which is a reasonable assumption for urbanized areas.
